# Impact of Large Gate Voltages and Ultrathin Polymer Electrolytes on Carrier Density in Electric-Double-Layer-Gated Two-Dimensional Crystal Transistors

**DOI:** 10.1021/acsami.2c13140

**Published:** 2023-03-16

**Authors:** Shubham Sukumar Awate, Brendan Mostek, Shalini Kumari, Chengye Dong, Joshua A. Robinson, Ke Xu, Susan K. Fullerton-Shirey

**Affiliations:** † Department of Chemical and Petroleum Engineering, University of Pittsburgh, Pittsburgh, Pennsylvania 15260, United States; ‡ Department of Materials Science and Engineering, The Pennsylvania State University, University Park, Pennsylvania 16802, United States; § Center for 2D and Layered Materials and Center for Atomically Thin Multifunctional Materials, The Pennsylvania State University, University Park, Pennsylvania 16802, United States; ∥ Two-Dimensional Crystal Consortium, The Pennsylvania State University, University Park, Pennsylvania 16802, United States; ⊥ School of Physics and Astronomy, Rochester Institute of Technology, Rochester, New York 14623, United States; # Microsystems Engineering, Rochester Institute of Technology, Rochester, New York 14623, United States; % Department of Electrical and Computer Engineering, University of Pittsburgh, Pittsburgh, Pennsylvania 15260, United States; △ School of Chemistry and Materials Science, Rochester Institute of Technology, Rochester, New York 14623, United States

**Keywords:** electric double layer, finite element modeling, polymer electrolyte, two-dimensional materials, Nernst−Planck−Poisson, drift diffusion, thin electrolyte, quantum capacitance

## Abstract

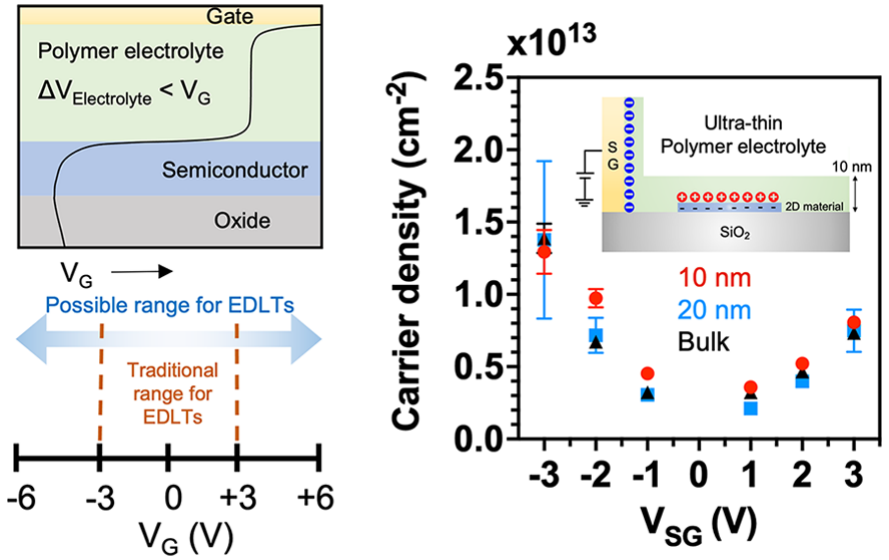

Electric-double-layer (EDL) gating can induce large capacitance densities (∼1–10 μF cm^–2^) in two-dimensional (2D) semiconductors; however, several properties of the electrolyte limit performance. One property is the electrochemical activity which limits the gate voltage (*V*_G_) that can be applied and therefore the maximum extent to which carriers can be modulated. A second property is electrolyte thickness, which sets the response speed of the EDL gate and therefore the time scale over which the channel can be doped. Typical thicknesses are on the order of micrometers, but thinner electrolytes (nanometers) are needed for very-large-scale-integration (VLSI) in terms of both physical thickness and the speed that accompanies scaling. In this study, finite element modeling of an EDL-gated field-effect transistor (FET) is used to self-consistently couple ion transport in the electrolyte to carrier transport in the semiconductor, in which density of states, and therefore quantum capacitance, is included. The model reveals that 50 to 65% of the applied potential drops across the semiconductor, leaving 35 to 50% to drop across the two EDLs. Accounting for the potential drop in the channel suggests that higher carrier densities can be achieved at larger applied *V*_G_ without concern for inducing electrochemical reactions. This insight is tested experimentally via Hall measurements of graphene FETs for which *V*_G_ is extended from ±3 to ±6 V. Doubling the gate voltage increases the sheet carrier density by an additional 2.3 × 10^13^ cm^–2^ for electrons and 1.4 × 10^13^ cm^–2^ for holes without inducing electrochemistry. To address the need for thickness scaling, the thickness of the solid polymer electrolyte, poly(ethylene oxide) (PEO):CsClO_4_, is decreased from 1 μm to 10 nm and used to EDL gate graphene FETs. Sheet carrier density measurements on graphene Hall bars prove that the carrier densities remain constant throughout the measured thickness range (10 nm–1 μm). The results indicate promise for overcoming the physical and electrical limitations to VLSI while taking advantage of the ultrahigh carrier densities induced by EDL gating.

## Introduction

Iontronic devices use ions to control the electronic properties of semiconducting materials,^1,2^ with large capacitance densities demonstrated in two-dimensional (2D)^3^ and organic semiconductors^4^ (∼1–10 μF cm^–2^). The mechanisms that govern iontronics can be broadly divided into two categories: electrochemical and electrostatic. Devices that rely on *electrochemistry* involve redox reactions at the electrolyte/channel interface, or inside the semiconductor, to modulate the carrier density in the channel materials.^4,5^ These include organic electrochemical transistors (OECTs),^6,7^ conductive-bridge memory (CBRAM),^8,9^ electrochemical random access memory (ECRAM),^10^ and ion intercalation devices.^11−13^ In contrast, iontronic devices rely on *electrostatic* interactions to modulate the carrier density by forming an EDL at the electrode/electrolyte and electrolyte/channel interfaces with no transfer of electrons (i.e., no electrochemistry). Included in this category are electric-double-layer transistors (EDLTs), which are FETs in which the gate oxide is replaced by an electrically insulating but ionically conductive electrolyte. While such a device could also undergo electrochemical reaction under sufficiently large gate voltage,^11,14−16^ in this study we consider EDLTs as ionically gated FETs for which electrochemical reactions are absent. As Leighton et al. highlights, this tends to be true for densely packed channel materials that are nonpermeable to ions.^5^ EDLTs and electric-double-layer capacitors (EDLCs) are widely studied for their applications as supercapacitors, sensors, flexible and printed electronics, and nonvolatile and resistive switching devices.^17,18^ EDLTs have also been employed to explore ferromagnetism, superconductivity, metal–insulator transition and light–matter interactions in 2D layered materials, organic semiconductors, and oxide semiconductors.^3,19−23^

**Figure 1:**
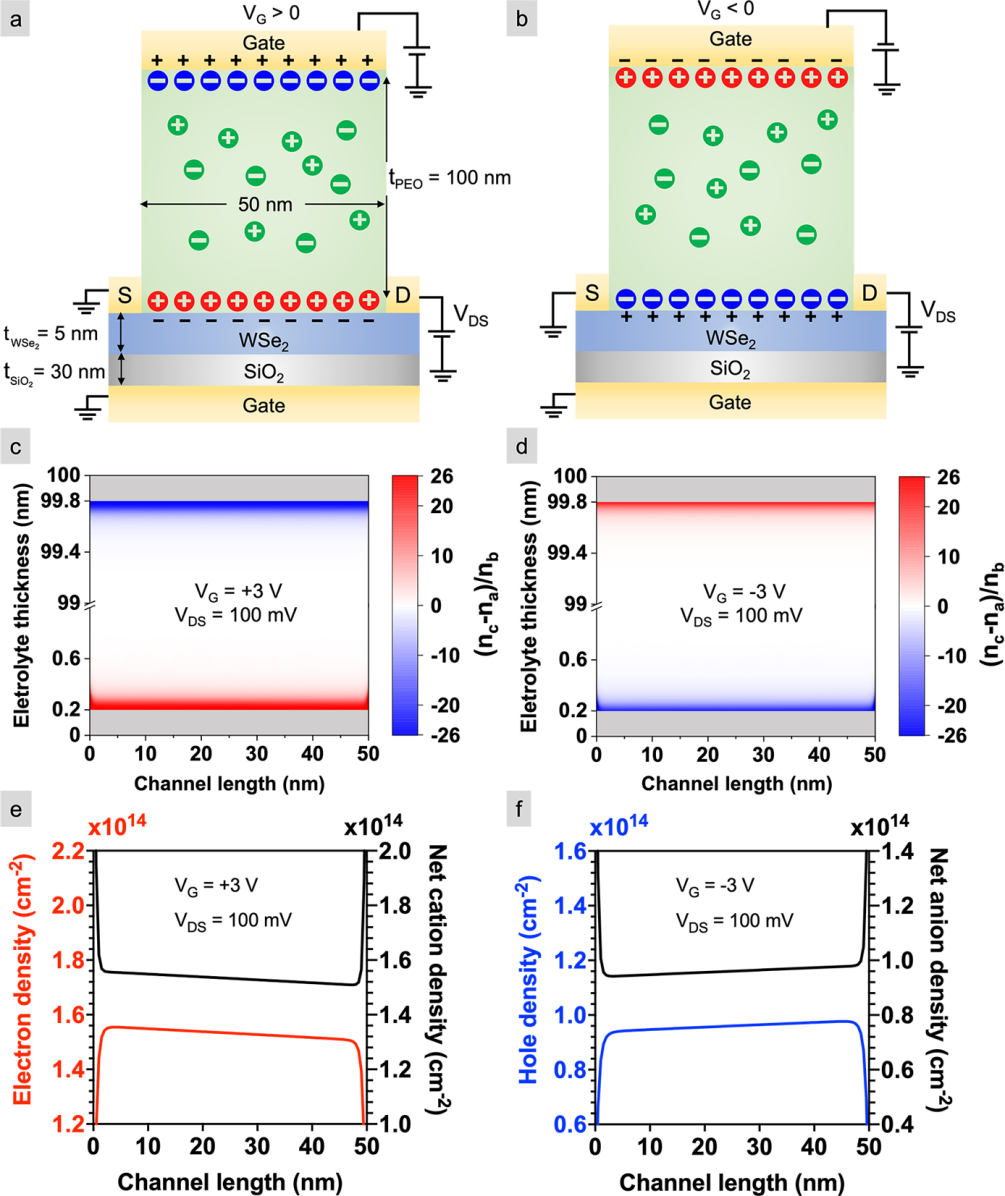
Device schematics and calculated net ion and carrier densities. EDL formation at the interfaces at (a) *V*_G_ > 0 and (b) *V*_G_ < 0. Net normalized ion concentration (*n*_c_ – *n*_a_)/*n*_b_ at the interfaces at (c) *V*_G_ = +3 V and (d) −3 V. Stern layers are indicated in gray at the top and bottom of the electrolyte. Sheet carrier densities and net ion densities at (e) *V*_G_ = +3 V and (f) −3 V. WSe_2_ thickness = 5 nm and *E*_gap_ = 1.25 eV.

In an EDLT, when no gate voltage is applied, the cations and anions are uniformly distributed in the electrolyte. When a positive gate voltage is applied (*V*_G_ > 0), anions drift toward the gate and cations to the semiconductor, forming an anionic EDL at the electrolyte/gate interface and cationic EDL at the electrolyte/semiconductor interface. The accumulation of cations at the semiconductor surface results in n-type doping, as shown in Figure 1a. When the polarity of the gate voltage is reversed (i.e., *V*_G_ < 0), the semiconductor is doped p-type, as shown in Figure 1b. The doping density in the channel is proportional to the applied gate voltage, where most of the potential drops across the two EDLs and very little potential drops across the charge neutral bulk of the electrolyte.

The primary motivation for using EDL gating is the large capacitance density (∼1–10 μF cm^–2^), which is ∼5–10 times higher than the traditional high κ dielectrics.^4,24,25^ Essentially, the EDL at the semiconductor surface acts as an extremely thin capacitor (thickness <1 nm), giving rise to high capacitance densities. This gating mechanism induces high sheet carrier densities (10^13^–10^14^ cm^–2^) in various organic and inorganic semiconductors.^3,26^ EDL gating is also used in fundamental research of low-dimensional materials. For example, this technique has been effective in controlling light scattering and emission,^27,28^ surface plasmon resonance,^22,23,29^ and the transition temperature for superconductivity^19,30,31^ in layered materials including graphene, 2D metals, and transition-metal dichalcogenides (TMDs).

The EDL capacitance and carrier densities in the semiconductor depend on the applied gate voltage. Because the electrolyte contains mobile ions, it can undergo electrochemical reactions when the voltage produces a field that is large enough to induce a redox reaction. Electrochemical reactions can be undesirable in EDLTs because they can induce heteroatom intercalation or change the composition of the electrolyte—both of which would interfere with investigating the fundamental properties of a chemically well-defined material.

In metal–insulator–metal (MIM) structures, all of the applied potential drops between two metals. However, in a FET structure, the applied voltage drops across a metal–insulator–semiconductor stack where the field distribution will be different than an MIM. Nonetheless, the maximum gate voltage applied to EDLTs is typically limited to ±3 V (i.e., electrochemical window^2^) as the standard reduction potentials (*E*_0_) for the components in the electrolyte is around 3 V (e.g.,  is −3.05 V vs standard hydrogen electrode (SHE)).^32−35^ The electrochemical window limits the maximum gate voltage that can be applied, thereby limiting the maximum extent to which carriers can be modulated. Surprisingly, there are reports of EDLTs with *V*_G_ extending to 5 V^13,36^ and even 7 V^19^ at room temperature, with no electrochemical signatures reported, which is 2 to 4 V beyond the commonly assumed electrochemical window. Such observations require further investigation to understand how the voltage changes with distance across the electrolyte and semiconductor in an ion-gated FET.

In addition to the performance constraints imposed by *V*_G_, another constraint—this one impacting the speed of the EDL response—is the ionic mobility of solid polymer electrolytes, which is orders of magnitude lower than electrons and holes.^37−39^ The polarization response time is quantified as *R*_B_*C*_E_, where *R*_B_ is the electrical resistance of the electrolyte and *C*_E_ is the EDL capacitance. Because *R*_B_ is proportional to the thickness of the electrolyte, decreasing the thickness will decrease the time constant which corresponds to faster polarization.^41^ The thickness of commonly studied polymer electrolytes are in the range of 0.2–1 μm,^40^ which is at least 2 orders of magnitude higher than traditional gate oxides.^40^ Thus, the polarization response is slow—on the order of few hundreds of milliseconds to a few seconds for ∼1 μm PEO:CsClO_4_.^42−45^ Decreasing the electrolyte thickness from 1 μm to 10 nm will decrease the *R*_B_*C*_E_ time constant by ∼100×, making electrolytes potentially more suitable for high-speed applications. However, it is essential to first show whether or not polymer electrolytes scaled to tens of nanometers can induce large carrier densities (10^13^–10^14^ cm^–2^) in semiconductors. Without such high doping densities, speed becomes less relevant.

Although computational approaches have been employed to better understand EDLTs, only a few studies account for the coupled physics between the ion and carrier transport.^46,47^ Paletti et al. used finite element modeling to demonstrate an EDL Esaki diode by simulating the voltage-dependent equilibrium ion and carrier density profiles.^48^ Fathipour et al. demonstrated the formation of a p–i–n junction and quantified the ion and carrier concentrations near the contact regions.^49^ In both studies, the devices had a FET structure but were operated as p–i–n^49^ and Esaki^48^ diodes by applying a lateral electric field between source and drain. Also, both studies focused on steady-state carrier distributions under a fixed voltage and, as such, used the ion concentration at the electrolyte/semiconductor interface as a fixed charge input to calculate the steady-state carrier density in the channel. Ueda et al. simulated an EDLT by self-consistently coupling ion and carrier transport using finite difference method, and their work focused on explaining ambipolar behavior in WSe_2_ using a gate voltage comparable to the band gap energy.^47^

In this study, we use finite element modeling to simulate the dynamic response of ion and carrier transport in an EDLT to solve for (1) the voltage distributions across the electrolyte and the semiconductor and (2) ion and carrier densities as a function electrolyte thickness. By self-consistently coupling ion and charge transport, a more accurate estimate of FET charge density is achieved compared to assuming an MIM structure for which no potential drops across the metal electrodes. Importantly, the results show that 50 to 65% of the applied potential drops across the semiconductor, leaving 35 to 50% to drop across the two EDLs. This result suggests that higher charge densities can be achieved with larger gate voltages while avoiding electrochemistry. We verify this experimentally by measuring the carrier densities via Hall effect on graphene FETs at gate voltages up to ±6 V. In addition, the thickness scalability of solid polymer electrolytes is modeled down to 10 nm in both top- and side-gated geometries, showing that carrier density is independent of thickness—as expected. The results are validated experimentally using a graphene FET with PEO:CsClO_4_ thinned to 10 nm. No significant change is observed between the carrier densities using ultrathin and bulk electrolytes, suggesting the possibility of scaling electrolytes to VLSI-relevant thicknesses.

## Results and Discussion

### Carrier Density at Large (±6 V) Gate Voltages

To model the voltage distribution across both the electrolyte and semiconductor, the concentration distribution of all charged species (i.e., ions in the electrolyte and carriers in the semiconductor) is calculated self-consistently. Here, a top-gated FET structure (Figures 1a,b) is modeled, where few-layer WSe_2_ (thickness = 5 nm and *E*_gap_ = 1.25 eV) is gated using a solid electrolyte (dielectric constant ϵ_PEO_ = 10 and ion concentration *n*_b_ = 1000 mol/m^3^). Note that *n*_b_ and ϵ_PEO_ used for modeling (see the Methods section, Table 1) are chosen to match experimental values.^41^ The modified Poisson–Nernst–Planck (mPNP) equations are solved simultaneously with the drift-diffusion equations to numerically calculate the concentration of ions within the EDL, the distribution of electrons and holes in the semiconductor, and the distribution of electric potential throughout the device geometry (see the Methods section). A Fermi–Dirac distribution of the energy states and the parabolic dispersion relation is used to account for the density of states in the carrier density calculations (i.e., quantum capacitance is accounted for in this model). The concentration of ions and carriers at two gate voltages, *V*_G_ = +3 and −3 V, are calculated for *V*_D_ = 100 mV. Surface plots of the net normalized ion concentration (*n*_c_ – *n*_a_)/*n*_b_ as a function of channel length (*x*) and electrolyte thickness (*y*) for *V*_G_ = +3 and −3 V are shown in Figures 1c and 1d, respectively, where *n*_c_, *n*_a_, and *n*_b_ are cation, anion, and bulk ion concentrations, respectively. In the regions less than 1 nm from the electrolyte/semiconductor and gate/electrolyte interfaces, the ions accumulate ∼26 times more than the bulk concentration, creating subnanometer gap capacitors.

**Table 1:** Electronic and Physical Properties Used for Finite Element Simulations

parameter	symbol	value	ref
electron affinity of WSe_2_	χ_WSe_2__	3.7 eV	(66)
band gap	*E*_gap_	1.25 eV	(67)
electron mobility	μ_n_	43 cm^2^/(V s)	(47)
hole mobility	μ_p_	84 cm^2^/(V s)	(47)
relative permittivity of WSe_2_	ε_WSe_2__	7.25	(68)
relative permittivity of electrolyte	ε_PEO_	10	(69)
relative permittivity of SiO_2_	ε_SiO_2__	8	(70)
Stern layer thickness	a	0.2 nm	
bulk ion concentration	*n*_b_	1000 mol/m^3^	

In addition to surface plots in Figures 1c,d, the net ion densities and charge carrier densities are shown in Figures 1e,f as a function of channel length. The average electron and hole densities are ∼1.5 and ∼0.9 × 10^14^ cm^–2^ at *V*_G_ = +3 and −3 V, respectively, the same order of magnitude (10^13^–10^14^ cm^–2^) as previously reported experimentally using ionic liquids and solid polymer electrolytes.^42,45,47,50^ Moving from source to drain, the observed decrease (increase) in the electron (hole) density arises from the lateral electric field caused by a nonzero *V*_DS_ (100 mV). The generation of the carriers in the channel as a result of ion accumulation confirms that the ions in the electrolyte and the carriers in the semiconductor are electrostatically coupled by means of the electric potential. We refer to this as the mPNP+drift-diffusion model. Here, WSe_2_ is chosen as a representative 2D semiconductor; however, the model can be extended to other types of semiconductors including other 2D crystals, oxide semiconductors, and organic semiconductors by modifying the material properties listed in Table 1. We use an ion radius of 0.2 nm, which well approximates Cs^+^; carrier densities for additional ionic radii of 0.08 and 0.4 nm are reported in the Supporting Information, Part 1.

**Figure 2:**
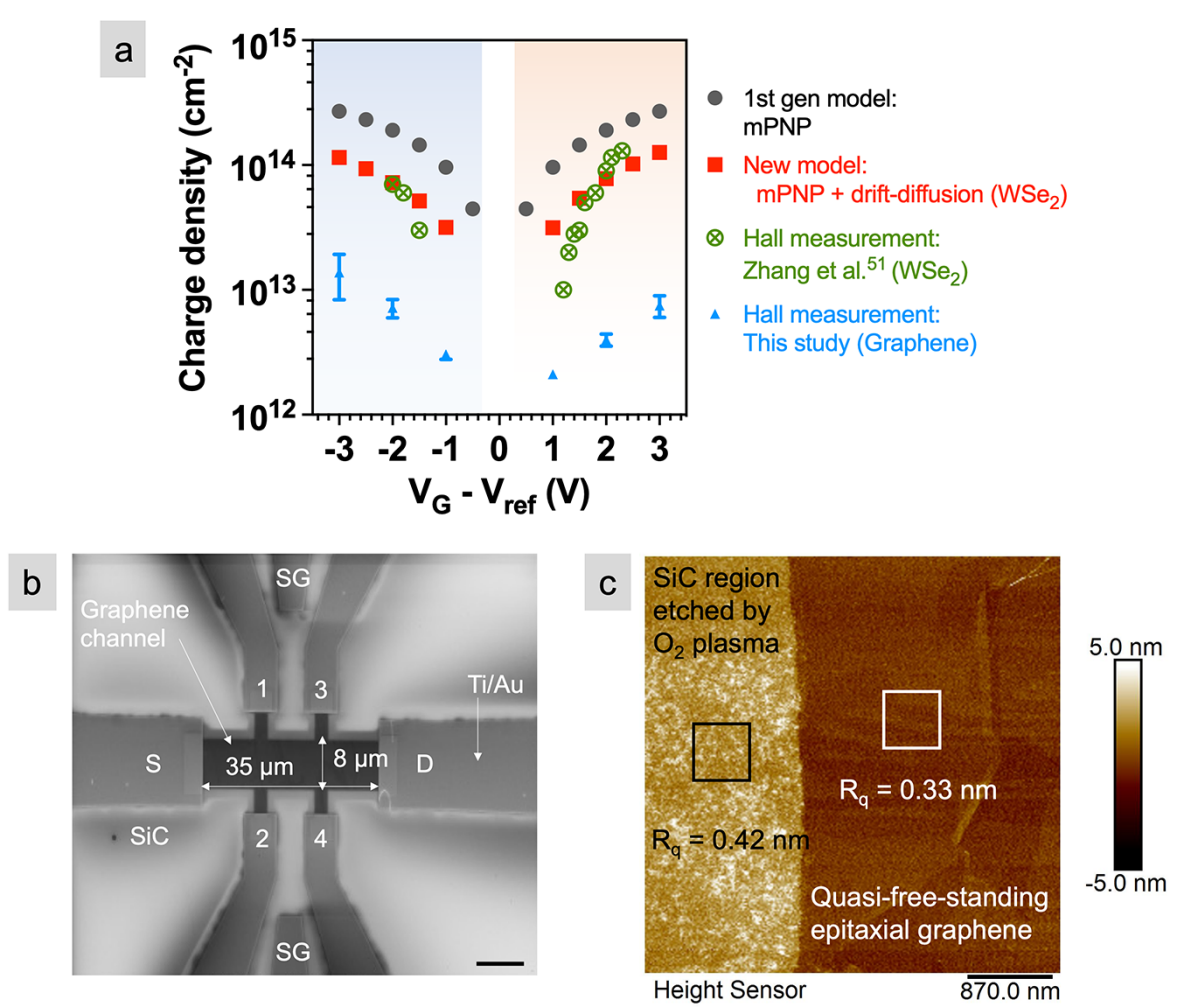
Calculations and measurements of electron and hole densities. (a) Charge densities calculated using the mPNP model and the mPNP+drift-diffusion model for WSe_2_ and measured by the Hall effect for WSe_2_ gated by an ionic liquid^51^ and QFEG gated using PEO:CsClO_4_. *V*_ref_ is zero except for the mPNP+drift-diffusion model where it represents the work function difference of −0.5 V between the Au contacts and WSe_2_ and Zhang et al.^51^ where it equals the threshold voltage of −1 V. The error bars for QFEG represent propagation of error in the measurement of Hall voltage. (b) SEM image of the Hall bar device before electrolyte deposition; the scale bar is 6 μm, and the length and width of the channel are 35 and 8 μm, respectively. The side gate is 5 μm wide and located 20 μm from the channel. (c) AFM topology scan of the lateral interface between graphene and SiC. The roughness (*R*_*q*_) is averaged over five 500 × 500 nm^2^ scans. Note that the SiC etched with oxygen plasma creates SiOx which is less dense than SiC; therefore, the etched region is topographically higher than the unetched region.

The sheet carrier densities calculated using the mPNP+drift-diffusion model on WSe_2_ are compared to experimental measurements via the Hall effect by Zhang et al.^51^ on monolayer WSe_2_ gated with ionic liquid and by us on quasi-free-standing epitaxial graphene (QFEG)^52,53^ gated using PEO:CsClO_4_ (Figure 2a). The model is in reasonable agreement with the experimental measurements by Zhang et al. on monolayer WSe_2_. Although it would be straightforward to attribute the difference between the model predictions for WSe_2_ and the experimental results on QFEG purely to the difference between the channel materials, we note that sheet carrier densities measured by the Hall effect spanning this entire range (10^12^–10^14^ cm^–2^) have been reported in the literature for both graphene and TMDs^19,20,45,51,54−59^ (Supporting Information, Part 2). These data are compiled in Figure S2 and Table S1.

Among all the data, our PEO:CsClO_4_-gated QFEG measurements give the smallest sheet densities. We cannot attribute this to device geometry because the sheet density increases with increasing gate-to-channel size ratio,^60−62^ and in our side-gated geometry, this ratio is already greater than one. One possible explanation relates to the 1 nm of resist residue that covers the graphene channel from the lithography process, as measured by AFM (Figure 2c). Removing this residue will increase the gating strength of EDL-gated FETs by 247%, as we showed previously.^63^

**Figure 3:**
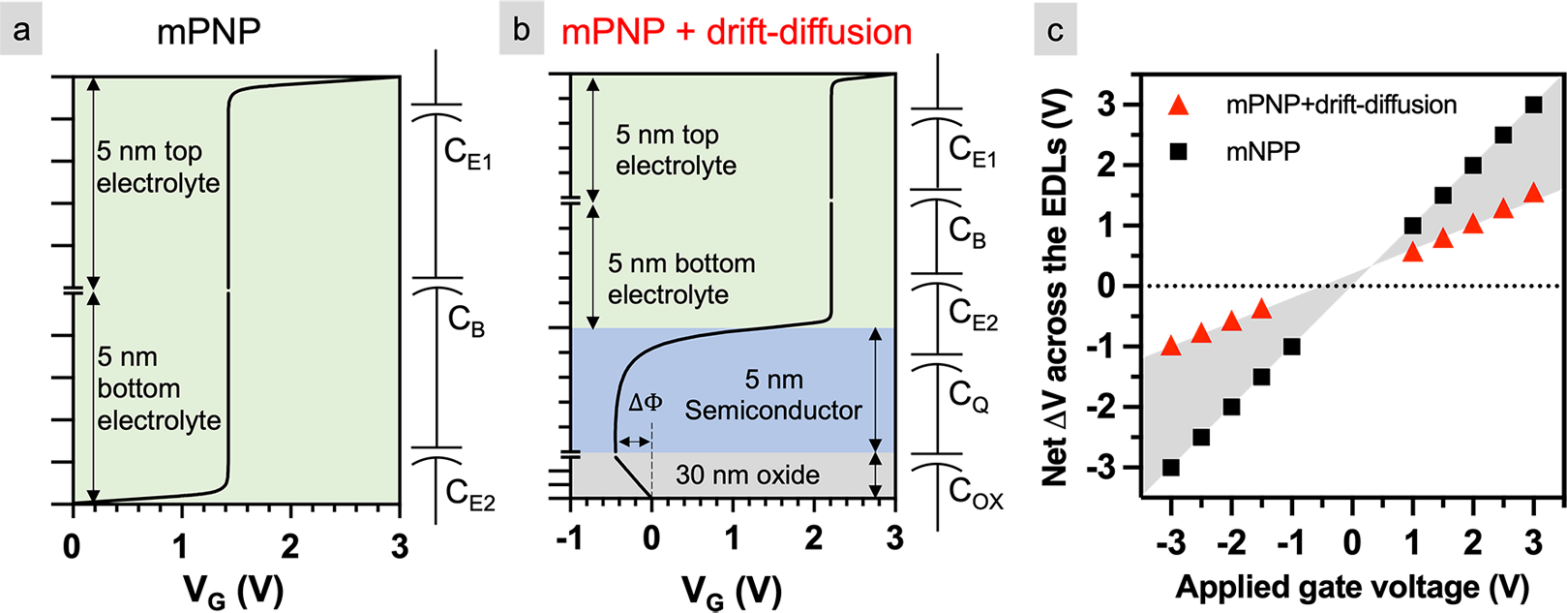
Voltage distribution in an EDLT: Voltage drop across (a) a MIM stack using the mPNP model and (b) a metal/electrolyte/semiconductor (MIS) stack using the mPNP+drift-diffusion model, both at *V*_G_ = +3 V. For clarity, the potential drop in the electrolyte is shown only in the region where it changes (i.e., nm from the semiconductor and the gate). The equivalent circuits show capacitors in series for the MIM and MIS stacks. *C*_E1_ and *C*_E2_ are the EDL capacitances. *C*_B_, *C*_Q_, and *C*_OX_ are the bulk electrolyte, quantum, and oxide capacitances, respectively. (c) Δ*V* across the EDLs as a function applied gate voltage for mPNP and mPNP+drift-diffusion model. The shaded region represents the voltage dropped across the semiconductor.

The sheet carrier densities calculated using the new model are also compared to other models where the electrolyte is modeled within an MIM structure.^48−50,64,65^ In the MIM, only the modified Nernst–Planck and Poisson’s equations are used to calculate the ion density inside the EDL (referred here as mPNP model), which can then be used as a fixed-charge input to calculate the carrier density inside the semiconductor. The MIM approximates the metal/electrolyte/semiconductor stack by assuming that the dense sheet of ions at the electrolyte/semiconductor interface acts like a metal and blocks the field from the semiconductor. However, the mPNP model overestimates the carrier densities by approximately 1 order of magnitude compared to the mPNP+drift-diffusion model at the same applied gate voltage (Figure 2a). The difference can be accounted for by considering the voltage distributions between the mPNP model applied to an MIM structure versus the mPNP+drift-diffusion model applied to the FET structure. As shown in Figure 3a, all the applied gate voltage, *V*_G_ = +3 V, drops across the EDLs (1.5 V at the top EDL and 1.5 V at the bottom EDL when the electrode areas are equal, i.e., *C*_E1_ = *C*_E2_ in Figure 3a) in the MIM configuration as described by the mPNP model. Irrespective of the electrode areas, the total voltage drop across the EDLs will always be equal to the total applied voltage in an MIM configuration.

In contrast, for the mPNP+drift-diffusion model with a semiconductor channel, the total voltage drop across the EDLs is smaller than the total applied voltage (Figure 3b) because part of the applied voltage drops across the generated carriers inside the semiconductor. Recall that quantum capacitance is included by accounting for the DOS in the mPNP+drift-diffusion model, as indicated by *C*_Q_ in the equivalent circuit (Figure 3b). To quantify the difference between the two models, we apply *V*_G_ in the range of −3 to +3 V in 0.5 V intervals and predict the voltage distribution across the entire geometry using both the models. The Δ*V* across the EDLs as a function of *V*_G_ is shown in Figure 3c. For mPNP+drift-diffusion model on WSe_2_, the Δ*V* across the EDLs is ∼50 to 65% smaller than the applied *V*_G_, highlighted by the shaded region in Figure 3c. Note that in the mPNP+drift-diffusion model (Figure 3b), the voltage in the bottom region of the semiconductor reaches ∼−0.5 V because of the built-in potential as a result of the work function difference (ΔΦ) between the contact metal (Au) and the semiconductor. This means that the overall voltage drop across the EDLs and the semiconductor is 0.5 V more than applied *V*_G_ = +3 V. Similarly, for *V*_G_ = −3 V, the voltage at the bottom of the semiconductor reaches ∼−2.5 V, thereby making the total potential drop across the EDLs and semiconductor less than the applied *V*_G_ = −3 V. Thus, the Δ*V* across the EDLs becomes zero at *V*_G_ = ∼− 0.5 V in Figure 3c (data in triangles) because of the built-in potential. Also note that the voltage through the back gate oxide goes to zero because the back gate is set to *V*_BG_ = 0 V.

The extent to which the potential drops in the electrolyte is critical to EDL-gated FETs because it dictates the maximum applied voltage that can be used to modulate the carrier density without inducing electrochemistry. As mentioned in the Introduction, there are several reports where the electrolyte gate voltage far exceeds what we know to be the electrochemical window. For example, Heidarlou and co-workers reported EDL gating of WSe_2_ FETs with PEO:CsClO_4_ using a gate voltage up to ±5 V.^36^ Shi and co-workers used a *V*_G_ = 7 V to gate WS_2_ using poly(ethylene glycol) (PEG):KClO_4_.^19^ Efetov and Kim et al. gated graphene using PEO:LiClO_4_ at gate voltages up to ±15 V for *T* < 250 K.^54^ The low temperature was used to kinetically arrest the Li^+^ and ClO_4_^–^ ions in the electrolyte to avoid electrochemical reactions. These studies report electrical characteristics that do not indicate electrochemistry, even though this voltage is large compared to the electrode potential of Li^+^ (−3.05 V vs SHE). Our modeling provides insight as to why this seemingly contradictory result can be true: the voltage drop across the electrolyte (i.e., the electrochemically active material) is smaller than the applied voltage. The implication is that it could be possible to create stronger than expected EDLs without inducing electrochemistry because larger applied voltages may not correspond to voltage differences across the electrolyte that are sufficient to induce redox reactions.

**Figure 4:**
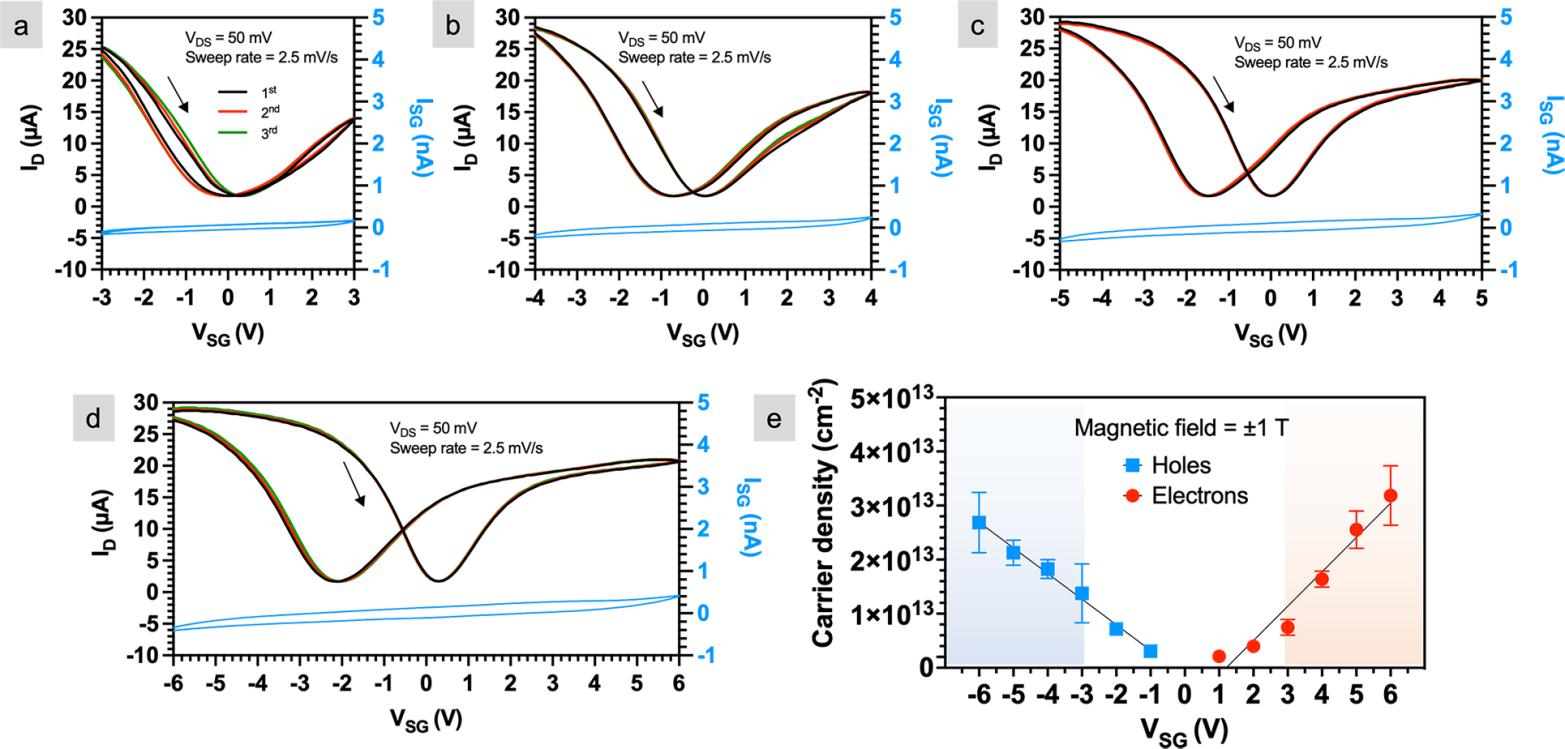
Electrical measurements at larger gate voltages. Drain current (*I*_D_) and gate leakage current (*I*_SG_) as a function of side gate voltage (*V*_SG_) of quasi-free-standing epitaxial graphene (QFEG) gated using bulk (∼1 μm thick) PEO:CsClO_4_ in gate voltage (*V*_SG_) range of (a) ±3, (b) ±4, (c) ±5, and (d) ±6 V. (e) Electron and hole densities as a function of *V*_SG_ measured via Hall effect measurements on a QFEG Hall bar structure using the same electrolyte. The error bar represents uncertainty as a result of propagation of error in the measurement of Hall voltage.

To experimentally check if it is possible to create stronger EDLs without inducing electrochemistry, the drain current (*I*_D_) and gate leakage current (*I*_SG_) were measured on a QFEG FET with ∼1 μm thick PEO:CsClO_4_, while the side gate was increased from ±3 to ±6 V. When electrochemical reactions occur, *I*_D_ will suddenly increase as *V*_G_ is swept.^13^ There can also be side reactions at the Au/electrolyte interface detectable by *I*_SG_, which may not significantly effect the transport properties of the channel.^13^ Specifically, *I*_SG_ will suddenly increase and slowly decay as the reaction transitions from a kinetically controlled regime to a transport-limited one. Before starting the measurement, a steady-state EDL was created by holding the gate voltage constant for 10 min, which far exceeds the time required to build the EDL.^42,45^ As expected, for *V*_SG_ in the range of ±3 V (Figure 4a), the starting value of the drain current and the hysteresis are similar for all three sweeps, and *I*_SG_ remains below ±5 nA. Both observations support electrostatic gating only.

Figures 4b, 4c, and 4d represent the *I*_D_ and *I*_SG_ when the gate voltage range is increased to ±4, ±5, and ±6 V, respectively. Similar to ±3, the transfer measurements are identical for all three repeats, and the gate leakage current remains below ±5 nA, suggesting primarily electrostatic interactions for *V*_SG_ up to ±6 V. In addition, no abrupt current changes are observed during the 10 min hold time—these data are reported in the Supporting Information, Part 3. This large voltage range for EDL-gated graphene FETs is not unexpected. Bediako and co-workers measured the electrochemical response of graphene sandwiched between h-BN using a Pt pseudoreference electrode and reported that the voltage required to intercalate lithium into graphene/h-BN interface using PEO:LiTFSI was >5 V (−2.75 vs Pt).^13^ Thus, on both the epitaxial graphene studied here and graphene on h-BN, these results show an electrochemical onset voltage larger than expected.

Hall effect measurements are made on QFEG to quantify the carrier densities at larger gate voltages. The Hall structures were gated using ∼1 μm PEO:CsClO_4_. Under the gate voltage range of *V*_SG_ = ±3 V, which is conventionally considered as the electrochemical limit in the absence of a reference electrode, the measured electron density was ∼0.75 × 10^13^ cm^–2^ and hole density was ∼1.4 × 10^13^ cm^–2^ (Figure 4e). At the largest gate voltages measured (*V*_SG_ = ±6 V), both the electron and hole densities increased to ∼3.1 × 10^13^ and ∼2.7 × 10^13^ cm^–2^, respectively. Thus, applying ±6 V enabled carrier density modulation by an additional Δ*n*_e_ = 2.3 × 10^13^ cm^–2^ (∼250%) for electrons and Δ*n*_h_ = 1.4 × 10^13^ cm^–2^ (∼100%) for holes compared to *V*_SG_ = ±3 V. Although mobility is not measured here, we previously reported the mobility of electrons (holes) in epi-graphene gated using PEO:LiClO_4_ as 480 (200) cm^2^/(V s) near the Dirac point at room temperature.^45^

### EDL Gating Using an Ultrathin Polymer Electrolyte

**Figure 5:**
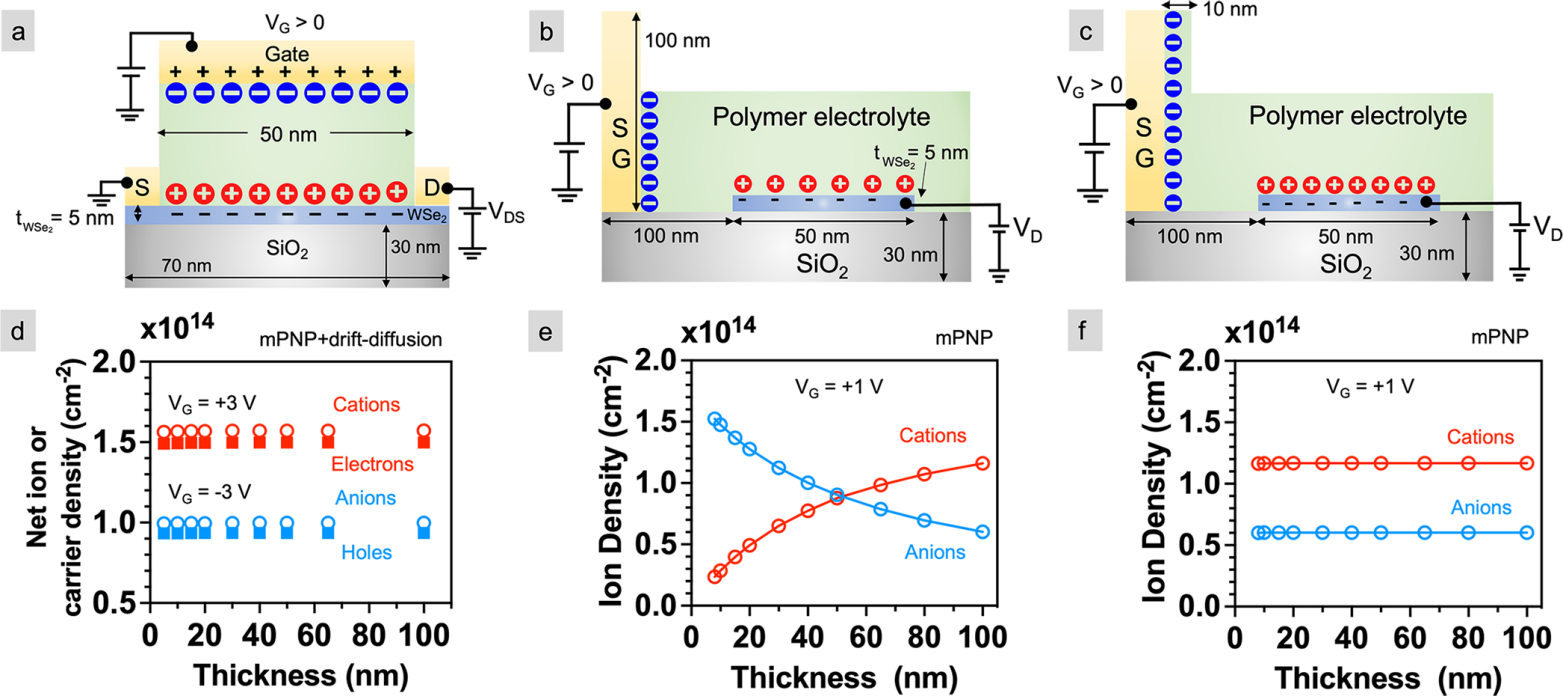
Calculated ion and carrier densities as a function of electrolyte thickness. Schematics of (a) top gate, (b) partially covered side gate, and (c) fully covered side gate COMSOL geometries. (d) Net ion density (empty circles) and sheet carrier density (filled squares) for *V*_G_ = ±3 V and *V*_DS_ = 100 mV for the model represented in (a). (e, f) Net ion density near the semiconductor/electrolyte interface (red empty circles) and gate/electrolyte interface (blue empty circles) at *V*_G_ = +1 V for the models represented in (b, c), respectively. Note that for the side gate geometry, the semiconductor channel is held at a constant potential of 100 mV, corresponding to the maximum voltage applied across the channel in experiments.

As mentioned in the Introduction, the thicknesses commonly used for EDL gating are in the range of 0.2–1 μm, which are incompatible with VLSI electronics—both due to physical thickness and speed. To improve the polarization response speed, the scalability of solid polymer electrolyte thickness and its impact on the carrier density are studied using numerical simulations and experimental measurements. EDLTs in three geometries are studied: one top-gated and two side-gated (Figure 5). In the side-gated structure, two arrangements are chosen to represent two possible scenarios of electrolyte films on the gate. In first side-gated geometry, the amount of gate in contact with the electrolyte is equal to the electrolyte thickness as shown in Figure 5b (referred to as partially covered side gate). In the second geometry, the amount of gate in contact with the electrolyte is a constant that is equal to height of the side gate as shown in Figure 5c (referred to as fully covered side gate). The results for the fully covered side gate geometry are validated experimentally by measuring the carrier density using an ultrathin solid polymer electrolyte.

Calculated ion and carrier densities at the electrolyte/semiconductor interface for a top-gated geometry (Figure 5a) for electrolyte thicknesses ranging from 5 to 100 nm at *V*_G_ = ±3 and *V*_D_ = 100 mV are shown in Figure 5d. The accumulated net ion density is essentially constant for all the thicknesses: ∼1.55 × 10^14^ and ∼1 × 10^14^ cm^–2^ for *V*_G_ = +3 and −3 V, respectively. Similarly, the induced electron and hole densities in the channel are also independent of the electrolyte thickness.

For the partially and fully covered side gate geometries (Figures 5b,c), only the mPNP equations are solved to numerically calculate the concentration of ions without the drift-diffusion equations. This is a limitation of modeling a side-gated geometry in 2D because the *V*_D_ and *V*_S_ cannot be applied simultaneously. The semiconductor channel is held at a constant potential of 100 mV, corresponding to the maximum voltage applied across the channel in experiments. In the partially covered side gate geometry, the cation density at the electrolyte/semiconductor interface decreases nonlinearly by 0.93 × 10^14^ cm^–2^ as the electrolyte thickness decreases from 100 to 5 nm (Figure 5e). Similarly, the anion density at the gate/electrolyte interface increases by the same amount. The trend in ion density can be explained by the change in the gate-to-channel length ratio. With the decrease in thickness, the gate-to-channel ratio decreases. When the channel is larger than the gate, the amount of charge buildup at the channel must be smaller than at the gate to maintain equivalent capacitance at both.^50^ A complementary trend occurs where the channel is smaller than gate. Because the channel length in our model is 50 nm, this dependence of ion density on the gate-to-channel ratio results in favoring ion buildup at the gate at thicknesses <50 nm and at the channel for thicknesses >50 nm. This is reflected in Figure 5e as a crossover in cation and anion densities.

In contrast, the fully covered side gate geometry (Figure 5c) demonstrates no such thickness dependence on ion density (Figure 5f). The gate is completely covered in 10 nm wide and 100 nm thick electrolyte as the remainder of the electrolyte varies in height. The constant gate-to-channel ratio means that the ion density is unaffected by thickness—the same as the top-gated geometry. These findings inform that the electrolyte can be made extremely thin (<10 nm) without significantly compromising the carrier density as long as the gate is coated in electrolyte, which is likely given that the polymer chains are entangled with the neighboring chains creating a continuous thin film.

**Figure 6:**
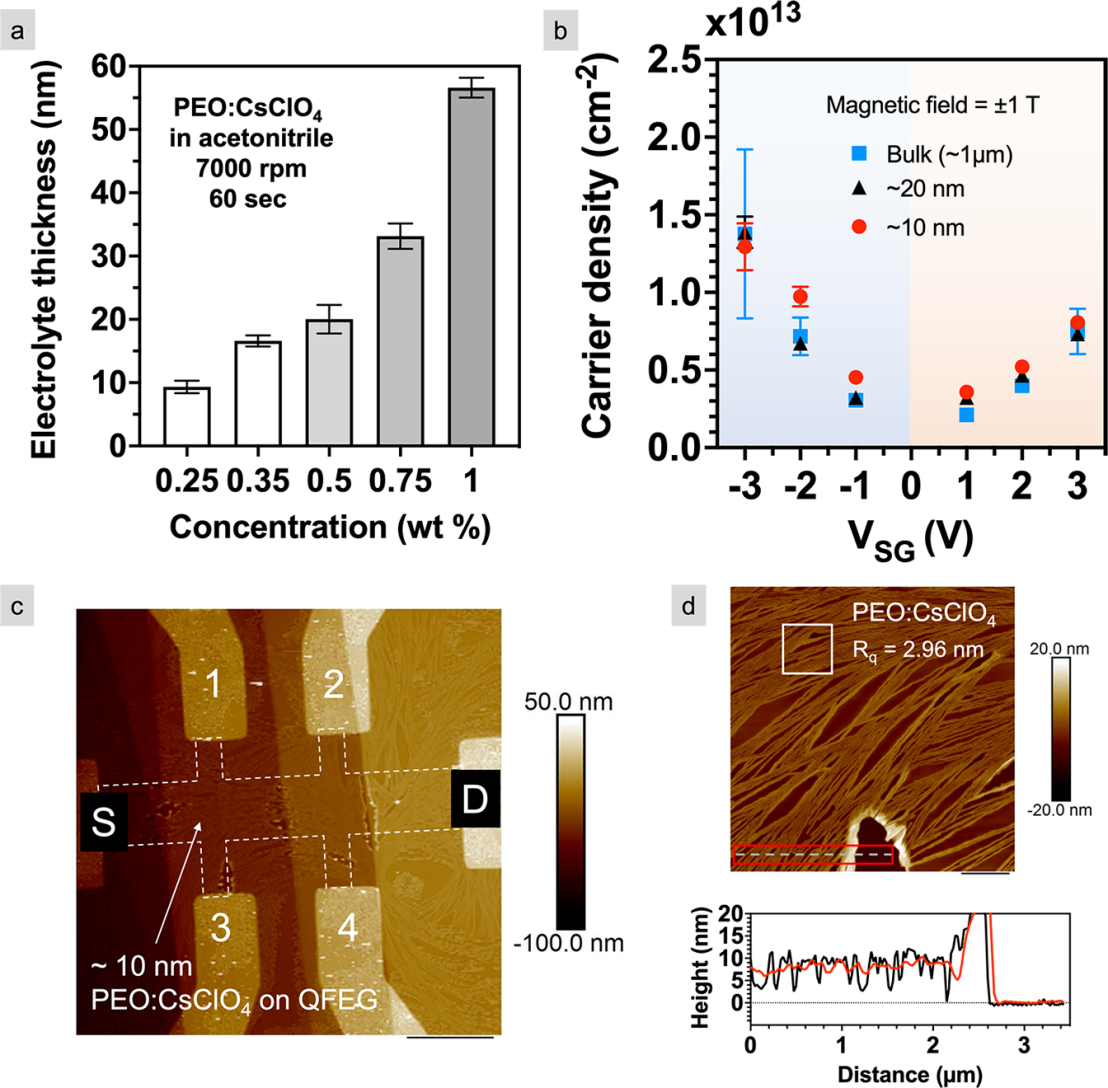
Electric-double-layer gating using an ultrathin polymer electrolyte. (a) Electrolyte thickness as a function of wt % of PEO:CsClO_4_ in acetonitrile. Error bars represents one standard deviation from the mean height of five measurements. (b) Graphene FET sheet carrier density as a function of gate voltage using ∼10, 20, and 1 μm thick PEO:CsClO_4_. The error bar represents uncertainty as a result of propagation of error in the measurement of Hall voltage. (c) AFM topography image of the ∼10 nm film on QFEG Hall structure. The dashed lines outline the channel shape underneath PEO:CsClO_4_. The scale bar is 8 μm. (d) Thickness characterization using magnified AFM image on the QFEG channel. A line scan (solid black line) and step height (solid red line) confirm an electrolyte thickness of ∼10 nm. The scale bar is 1 μm.

Experimentally, ultrathin PEO:CsClO_4_ films are prepared on SiO_2_ by spin-coating solutions of PEO:CsClO_4_ in acetonitrile. To adjust the thickness, solution concentrations were varied from 0.25 to 1 wt %, while holding the spin speed fixed at 7000 rpm. The spin coating was performed for 1 min in an Ar-filled glovebox, followed by annealing at 80 °C for 3 min to remove the excess solvent. A step edge is created between the PEO:CsClO_4_ and SiO_2_ by making a scratch in the film, and the thickness, ranging from 10–55 nm, is measured across the step edge using AFM (Figure 6a).

QFEG Hall structures are spin coated with ∼10 and 20 nm PEO:CsClO_4_. Surface characterization of the ultrathin electrolyte is performed using AFM. Despite the small thickness and lateral confinement created because of the contact metals, a continuous electrolyte film is observed as shown in Figure 6c. For the thinnest continuous film, the average thickness is ∼10 nm as measured across a boundary between bare channel and electrolyte in a zoomed-in AFM topography image (Figure 6d). The average surface roughness is ∼3 nm. Additional surface characterization for continuous 10–55 nm electrolyte films is reported in the Supporting Information, Part 4. Crystalline structures are detected on the ∼10 and 20 nm electrolyte, which indicates that the electrolyte is semicrystalline. Conversely, electrolytes with thickness >20 nm are smoother and have uniform coverage across the entire spin-coated area.

To assess whether scaling the electrolyte thickness to tens of nanometers compromises the ability to induce charge in the channel, the experimentally measured carrier densities using the ultrathin electrolytes are compared with those measured using the bulk electrolyte. The hole (electron) density using the bulk electrolyte is ∼1.4 × 10^13^ cm^–2^ (∼0.8 × 10^13^ cm^–2^) for *V*_SG_ = −3 (+3) V (Figure 6b). 10 and 20 nm PEO:CsClO_4_ give similar carrier densities to the bulk throughout the entire *V*_SG_ range and are comparable with our previous report (electron densities of (0.2–1) × 10^13^ cm^–2^ in graphene) using an ∼1 μm electrolyte. Although not measured, the carrier density change at higher gate voltages (e.g., ±6 V) using an ultrathin electrolyte is expected to be similar to densities reported in Figure 4d using bulk electrolyte. Note that the large error in the hole density at *V*_SG_ = −3 V for the bulk electrolyte is a result of a lower drain current (*I*_D_ = 2 μA) used during the Hall measurements (Supporting Information, Part 4). To correct this issue, *I*_D_ = 10 μA was applied during the Hall measurements for the 10 and 20 nm electrolyte, resulting in smaller error bars. Additional information about the time-dependent Hall voltage measurement for three different electrolyte thicknesses is given in the Supporting Information, Part 5.

Nearly equivalent carrier densities between ultrathin and bulk electrolytes are promising because the R_B_C_E_ time constants associated with EDL formation and dissipation will decrease with decreasing electrolyte thickness. While this scaling in speed does not apply to the side-gated geometries investigated here because the gate-to-channel distance is independent of electrolyte thickness, it will apply to a top-gate geometry where gate to channel distance is the electrolyte thickness, which would be needed for VLSI.

## Conclusions

Finite element modeling is used to calculate ion and carrier densities for EDL-gated FETs by self-consistently solving the mPNP equations for ion transport, along with the drift-diffusion equations for transport in the semiconductor that accounts for density of states. The voltage distribution shows that 50 to 65% of the applied potential drops across the semiconductor, leaving 35 to 50% to drop across the two EDLs. This result suggests that higher carrier densities can be achieved at larger voltages without concern for inducing electrochemical reactions. Indeed, transfer measurements show repeatable *I*_D_–*V*_SG_ data without any sudden increase in the gate current, and Hall measurements extended to ±6 V increase the electron and hole densities while maintaining no signatures of electrochemistry. We expect this result to apply to any ion-gated FET for which the semiconductor is nonpermeable to ions.

In addition to measuring over a large voltage window, the solid polymer electrolyte is scaled to 10 nm. As expected, based on the physics of EDL gating, the steady-state capacitance density is independent of electrolyte thickness. Specifically, both top-gated and fully covered side-gated models give constant carrier densities in the thickness range of 5–100 nm. Here, we experimentally extend this ultrathin film exploration to side-gated, 2D crystal FETs. Electrolyte films with thicknesses down to ∼10 nm are achieved by spin coating PEO:CsClO_4_. Hall measurements confirm that the sheet carrier density remains constant throughout the measured thickness range (10 nm–1 μm). The added benefit of scaling is a faster polarization response because of the reduced R_B_C_E_ time constant, which is critical for VLSI. Both the large voltage window without electrochemistry and the ultrathin electrolyte scaling results are important for achieving strong electrostatic gate control in 2D FETs and demonstrating progress toward VLSI-relevant dielectric thickness and speed.

## Methods

### Finite Element Modeling

Finite element modeling was performed using COMSOL Multiphysics ver. 5.5. The ion transport in the electrolyte, the electron and hole transport in the semiconductor, and the potential distribution in the entire geometry were calculated by simultaneously solving the modified Nernst–Planck equation (mNP), the drift-diffusion equations, and Poisson’s equation, respectively. The Fermi–Dirac distribution of the energy states and the parabolic dispersion relation for the density of states were assumed in COMSOL when calculating the electron and hole density. Note that the electric potential across the entire device geometry was solved simultaneously to couple the accumulated ions at the electrolyte/semiconductor interface with the carriers in the semiconductor.

The mNP equation (eq 1) is described as
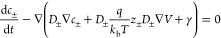
1where *c*_+_ (*c*_–_), *D*_+_ (*D*_–_), and *z*_+_ (*z*_–_) are concentrations (mol/m^3^), diffusivities (m^2^/s), and charge numbers of cations (anions) in the electrolyte, respectively. To calculate the ion concentration, the mNP equation was implemented only for the electrolyte region as a coefficient form partial differential equation. The term γ is the contribution from Kilic et al. to account for the size and therefore limit the packing density of the ions. The steric term (γ) in eq 1 is described as
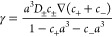
2where *a* is the diameter of the ion which is considered to be equal in this study for both the cations and anions.

The continuity equation for electron and hole current was solved where the electron and hole current density are described by the drift-diffusion equations
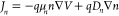
3a
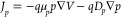
3bwhere μ_n_ (μ_p_) denote the mobility (m^2^/(V s)) of electrons (holes), *n* (*p*) are the electron (hole) concentrations (electrons or holes/m^3^), and *D*_n_ (*D*_p_) are the electron (hole) diffusivities (m^2^/s) as described by the Einstein relation. The values of the electronic and physical properties of the semiconductor and electrolyte are summarized in Table 1. All the charge transport from the mNP and the drift-diffusion equations was coupled with each other by the Poisson’s equations (eq 4) assuming the continuity of electric potential across the electrolyte semiconductor boundary.
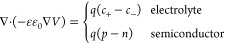
4where ε and *q* denote the relative permittivity and elementary charge, respectively. Equation 1 was solved using the general form PDE in the mathematics module. The current continuity equation and eqs 3a, 3b, and 4 were solved using the semiconductor module.

Metal contacts to the semiconductor were assumed to be purely ohmic. The source contact was grounded at all times whereas the drain and gate contacts were modeled as a boundary with nonzero electric potential. The potentials of the drain and gate contact were linearly increased from 0 to 100 mV and 0 to ±3 V, respectively. The potentials were held constant until steady state was reached. It is challenging to estimate the EDL thickness because of the continuous nature of the ion concentration even with the inclusion of the steric term and Stern layer. Therefore, to calculate the EDL ion densities, the net ion density, *n*_c_ – *n*_a_, along a cut line from the center of the electrolyte to the electrolyte/semiconductor interface was integrated using the trapezoidal rule. This approach ensured that all the ions in the EDL are accounted and the contribution from the ions in the charge neutral bulk electrolyte is excluded. Similarly, the majority carrier densities in the semiconductor were integrated from the backgate semiconductor–oxide interface to the electrolyte/semiconductor interface.

### Electrolyte Preparation and Deposition

The electrolyte PEO:CsClO_4_ was prepared by dissolving 80 mg of PEO (*M*_w_ = 110000 g/mol, Polymer Standards Service) and 21 mg of CsClO_4_ (99.9%, Sigma-Aldrich) in 10 g of anhydrous acetonitrile (Sigma-Aldrich) to make a 1 wt % solution with 20:1 ether oxygen to Cs^+^ molar ratio in an Ar-filled glovebox. The oxygen and water levels were maintained at O_2_ < 10 and H_2_O < 0.1 ppm. To prepare a bulk (∼1 μm) film, 40 μL of electrolyte solution was drop casted onto a 1 × 1 cm^2^ SiC substrate with graphene Hall structures, and the solvent was naturally evaporated for 5 min at room temperature. The sample was then annealed at 80^*o*^ C for 3 min to remove the remaining solvent and cooled naturally to room temperature before the electrical measurements. To prepare 10 and 20 nm films, the 1 wt % solution was diluted to 0.25 and 0.5 wt %, respectively; 80 μL of the electrolyte solution was spin coated at 7000 rpm for 60 s, and the solvent was naturally evaporated for 30 min at room temperature before performing the electrical measurements.

### Device Fabrication

Epitaxial graphene (EG) was grown by the sublimation of Si atoms from the Si face of 6H-SiC semi-insulating substrates (II–VI Advanced Materials Inc.) at 1800 °C. A Heidelberg Maskless Aligner MLA 150 was used to fabricate the EG Hall bars on SiC substrates where each of the devices was isolated utilizing nitrogen gas in a ULvac NE 550 etch tool. SEM was performed after completing the isolation layer. To enhance the adhesive strength of the metal on the EG/SiC surface, the contact regions were gently exposed to O_2_ plasma (descumming). Then the isolated devices were contacted with Ti (5 nm) and Au (20 nm) followed by another metallization step of Ti (5 nm) and Au (100 nm), which are deposited using an e-beam evaporator (Temescal). The metallization is done in two steps to avoid the electrode peeling off because of the thick metal near graphene during liftoff.

### Hall Carrier Density Measurements

Hall measurements were conducted in a Lakeshore cryogenic probe station with a vertical field superconducting magnet using a Keysight B1500A semiconductor parameter analyzer. The temperature of the sample stage was maintained at 300 K under the chamber vacuum of ∼5 × 10^–7^ Torr. During each measurement, the *V*_SG_ was applied and held constant for 900 s to allow ions to reach the steady state before starting the measurement. 2 μA of current for the bulk electrolyte and 10 μA for the 10 and 20 nm electrolyte were applied between the source and drain with the vertical magnetic field of 0 T, and the Hall voltage was monitored for 200 s. The magnetic field was gradually increased to +1 ± 0.01 T, and the Hall voltage was monitored for 200 s. The same sequence was repeated by reversing the polarity of the magnetic field to −1 ± 0.01 T. The measurements at 0 T were subtracted from the Hall voltages at *B* = ±1 T to eliminate the effect of the geometric asymmetry. The same measurement was repeated for all the reported *V*_SG_ values. The details of Hall measurements are reported in the Supporting Information, Part 5.

## Supporting Information Available

Impact of ion size on the calculated carrier density; literature comparison of ion-gated carrier densities in 2D materials; time-dependent *I*_D_ and *I*_SG_ for *V*_G_ = ±6 V; details of the Hall measurements; and surface characterization of additional ultrathin electrolyte films (PDF)
