# Supplementary material for: Impact of Large Gate Voltages and Ultrathin Polymer Electrolytes on Carrier Density in Electric-Double-Layer-Gated Two-Dimensional Crystal Transistors
Source: ACS Appl Mater Interfaces. 2023 Mar 16;15(12):15785–96. doi: 10.1021/acsami.2c13140 (PMC10064313; doi:10.1021/acsami.2c13140)
Supplement: Supplementary file 1 — am2c13140_si_001.pdf [file am2c13140_si_001.pdf]

Supporting Information: Impact of Large Gate Voltages and Ultra-thin Polymer Electrolytes on Carrier Density in Electric-double-layer-gated Two-dimensional Crystal Transistors

Shubham Sukumar Awate,[†] Brendan Mostek,[†] Shalini Kumari,[‡,¶] Chengye Dong,[§] Joshua A Robinson,[‡,¶,§] Ke Xu,[‖,⊥,†] and Susan K. Fullerton-Shirey*,[†,#,@]

†*Department of Chemical and Petroleum Engineering, University of Pittsburgh, Pittsburgh, Pennsylvania 15260, United States*

‡*Department of Materials Science and Engineering, The Pennsylvania State University, University Park, Pennsylvania 16802, United States*

¶*Center for 2D and Layered Materials and Center for Atomically Thin Multifunctional Materials, The Pennsylvania State University, University Park, Pennsylvania 16802, United States*

§*Two-Dimensional Crystal Consortium, The Pennsylvania State University, University Park, Pennsylvania 16802, United States*

‖*School of Physics and Astronomy and School of Chemistry and Materials Science, Rochester Institute of Technology, Rochester, New York 14623, United States*

⊥*Microsystems Engineering, Rochester Institute of Technology, Rochester, New York 14623, United States*

#*Department of Electrical and Computer Engineering, University of Pittsburgh, Pittsburgh, Pennsylvania 15260, United States*

@*Current address: 3700 O'Hara Street, Pittsburgh, Pennsylvania 15213, United States*

E-mail: fullerton@pitt.edu

Part 1: Effect of ion size on carrier density

Carrier density is calculated for three ion sizes (r = 0.08, 0.2 and 0.4 nm) at V_G = -3 to 3 V. It is expected that an ion with a smaller radius will have denser packing and therefore a higher EDL capacitance. As shown in Figure S1, the ion and carrier concentrations increase with decreasing ion size for all the gate voltages, as expected. For a five times smaller ion, the electron density increases by a factor of 3 at V_G = +3 V. For a similar decrease in ion size, the hole density increases by a factor of 2 at V_G = -3 V. The carrier densities predicted using the smallest ion (a = 0.08) are approximately one order of magnitude larger ($> 10^{14}$ cm^{-2}) than the typical experimentally achievable carrier densities using solid polymer electrolytes.[1]

Note that the size of the cations and anions are set to be equal to simplify the finite element calculations. In Figure S1, the ion concentrations in the electrolyte (filled squares) are always higher than the carrier concentrations in the semiconductor (open circles) because of the lower values of the carriers in the region near the contacts (Figure 1e and f).

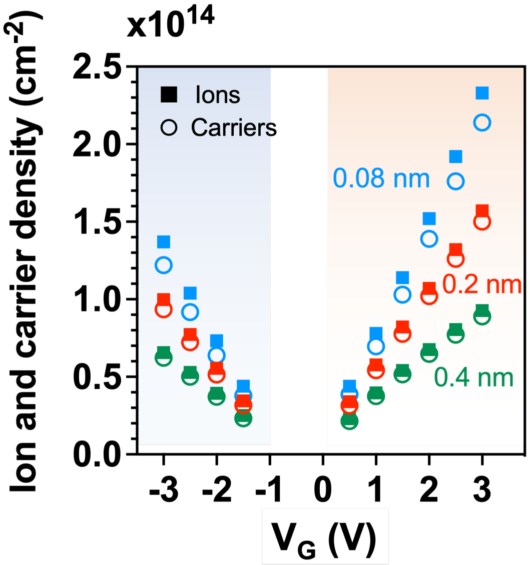

Figure S1: Ion density (filled squares) and sheet carrier density (empty circles) as a function of V_G and ion size. The electrolyte thickness is 100 nm and V_{DS} = 100 mV.

Part 2: Comparison of reported carrier densities using ion gating of 2D materials

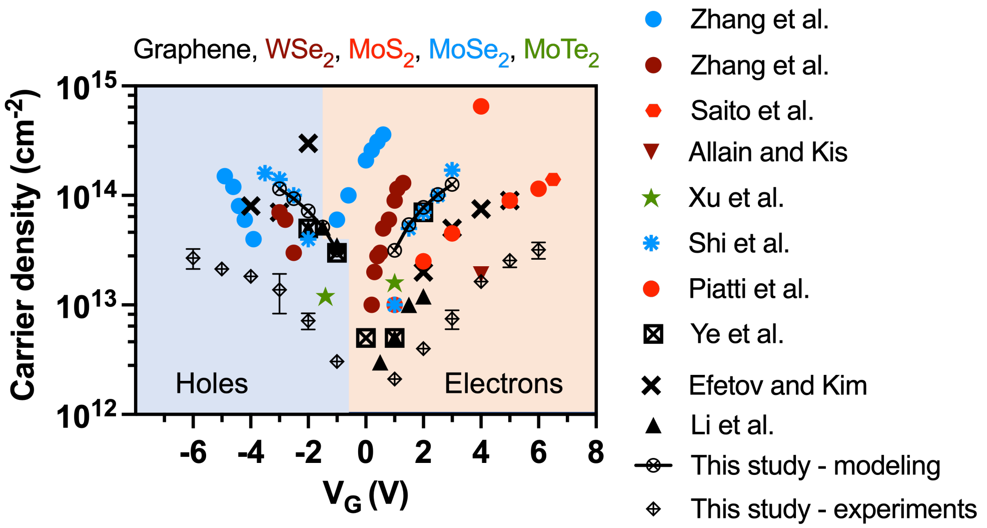

Figure S2: Electron and hole densities measured by Hall effect for a variety of graphene and TMD devices as a function of V_{SG}.

Carrier densities in a variety of 2D materials measured using Hall effect with different electrolytes are compared with the mPNP + drift-diffusion model on WSe_2 and experimentally measured carrier densities on epi-graphene in this study. Sheet carrier densities spanning the range of 10^{12} to 10^{14} cm^{-2} have been reported for both graphene and TMDs. Detailed values of densities, channel material and the type of electrolyte are reported in Table S1.

Table S1: Ion-gated carrier densities in Graphene and TMDs

**Material**	**Electrolyte**	**Carrier**	**V_G (V)**	**Density (cm^{-2})**	**Reference**
Graphene	PEO:$CsClO_4$	e	2−5	$2.00\times10^{13} - 9.00\times10^{13}$	Efetov and Kim[2]
Graphene	PEO:$CsClO_4$	h	-4−-2	$8.00\times10^{13} - 3.00\times10^{14}$	Efetov and Kim[2]
Graphene	DEME-TFSI	e	1−2	$5.00\times10^{12} - 7.00\times10^{13}$	Ye et al.[3]
Graphene	DEME-TFSI	h	-2−0	$5.00\times10^{13} - 5.00\times10^{12}$	Ye et al.[3]
Graphene	PEO:$LiClO_4$	e	0.5−2	$3.00\times10^{12} - 1.20\times10^{13}$	Li et al.[4]
Graphene	PEO:$LiClO_4$	h	-2−-1	$5.00\times10^{13} - 3.50\times10^{13}$	Li et al.[4]
MoS_2	BMPPD-TFSI	e	1−6	$1.00\times10^{13} - 1.15\times10^{14}$	Piatti et al.[5]
MoS_2	DEME-TFSI	e	6.5	1.40×10^{14}	Saito et al.[6]
MoS_2	DEME-TFSI	e	5	4.75×10^{14}	Zheliuk et al.[7]
$MoSe_2$	DEME-TFSI	e	1 − 3	$1.00\times10^{13} - 1.70\times10^{14}$	Shi et al.[8]
$MoSe_2$	DEME-TFSI	h	-3.5−-2	$1.60\times10^{14} - 4.00\times10^{13}$	Shi et al.[8]
$MoSe_2$	DEME-TFSI	e	-1−0.6	$6.00\times10^{13} - 3.60\times10^{14}$	Zhang et al.[9]
$MoSe_2$	DEME-TFSI	h	-4.9−-3.9	$1.50\times10^{14} - 4.0\times10^{13}$	Zhang et al.[9]
$MoTe_2$	PEO:$CsClO_4$	e	1	1.60×10^{13}	Xu et al.[10]
$MoTe_2$	PEO:$CsClO_4$	h	-1.4	1.20×10^{13}	Xu et al.[10]
WSe_2	PEO:$LiClO_4$	e	4	1.90×10^{13}	Allain and Kis[11]
WSe_2	DEME-TFSI	e	0.2 − 1.3	$1\times10^{13} - 1.3\times10^{14}$	Zhang et al.[9]
WSe_2	DEME-TFSI	h	-3−-2.5	$7.00\times10^{13} - 3\times10^{13}$	Zhang et al.[9]

Part 3: Electric-double-layer gating at larger gate voltages - time dependent channel and gate leakage current

Transfer measurements over a large gate voltage window (±6 V) resulted in highly repeatable I-V curves without any electrochemical signature in both the I_D and I_{SG} (Figure 4). As explained in the manuscript, the gate voltage was held constant for 10 min before starting the measurement to form a steady-state EDL. To check for an electrochemical signature during the hold time, I_D and I_{SG} are monitored at $V_{SG} = \pm6$ V for 10 min. During the charging of the EDL capacitor, I_D and I_{SG} showed typical exponential charging curves for an electrolytic capacitor (Figure S3a and b). All the repeated measurements overlap with

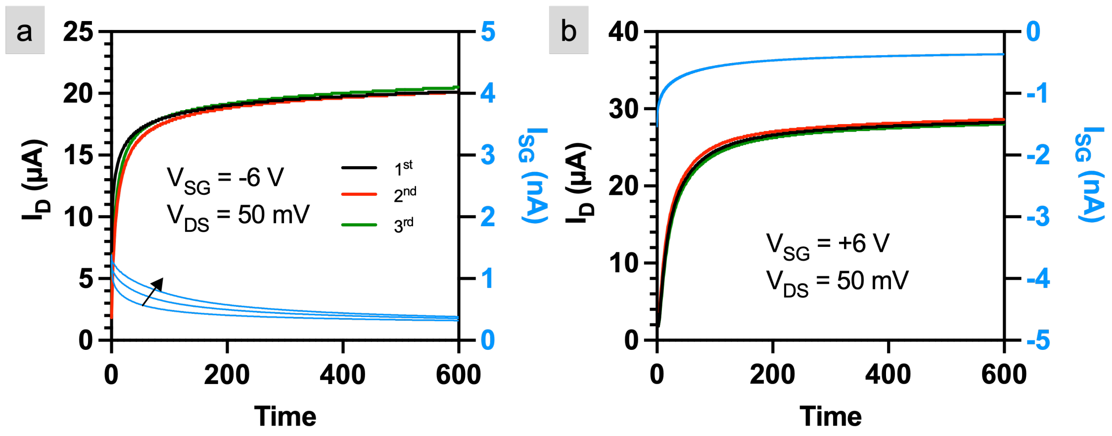

Figure S3: Channel and gate current as a function of time at larger gate voltages. Drain current, I_D, and gate leakage current, I_{SG}, when the side gate is held at (a) $V_{SG} = -6$ V and (b) +6 V for electrolyte thickness of ~ 1 μm and $V_{DS} = 50$ mV.

each other, suggesting the absence of electrochemical reactions during the three consecutive measurements. Note that for $V_{SG} = -6$ V (Figure S3(a)), there is a slight difference in the gate leakage current in three repeats at the start of the measurement, however, the difference is minimal and the data eventually overlap with time. This difference is not seen in the final transfer measurements reported in the manuscript (Figure 4d).

Part 4: Atomic force microscope (AFM) characterization of ultra-thin polymer electrolytes

As described in the manuscript, 10−55 nm electrolyte thin films are prepared by spin coating 0.25−1 wt% of PEO:$CsClO_4$ solution in acetonitrile on SiO_2. A scratch is made on the film to reveal the substrate, and surface characterization is performed using AFM at the boundary between the coating and SiO_2. The height of the coating is measured by taking a line scan from the AFM topography image. The root mean square roughness (R_q) of $\sim$ 10 nm coating was 4.9 nm. The roughness decreased to 3.5 nm with increase in thickness up to 20 nm and increased to 4.5 nm with increase in thickness up to 55 nm. Crystalline structures are detected for $\sim$ 10 and 20 nm electrolytes which indicates that ultra-thin polymer electrolytes are semi-crystalline, consistent with the phase diagram for the PEO electrolyte.[12–14]

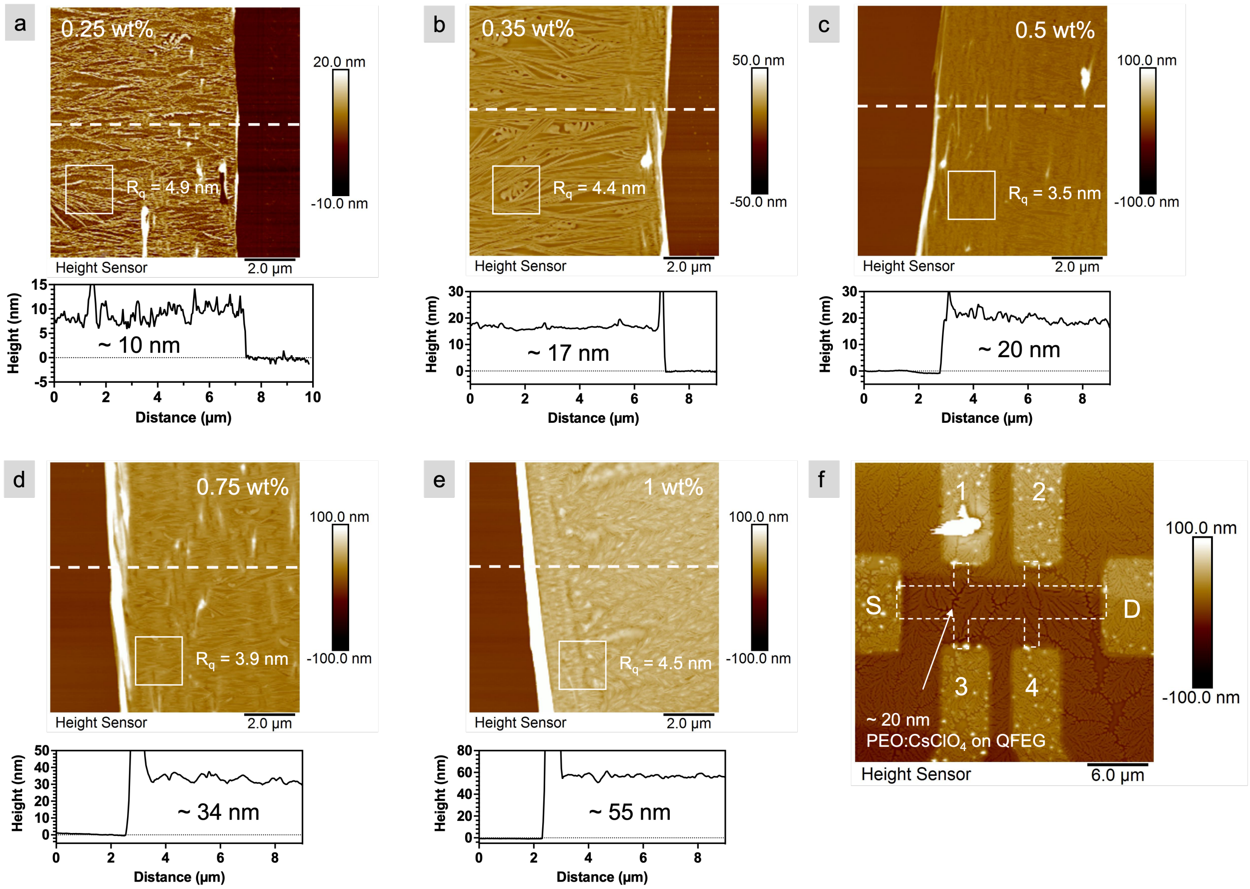

Figure S4: AFM characterization of ultra-thin electrolytes: AFM topography image and line scans of (a) 10, (b) 17, (c) 20, (d) 34 and (e) 55 nm electrolyte coatings. Roughness values are averages of five, 2 x 2 μm scans. Dashed white lines represent the location of the line scans. (f) AFM topography image of 20 nm coating on QFEG Hall structure. Dashed line outlines the channel shape underneath PEO:$CsClO_4$.

Part 5: Hall-effect measurements of carrier density gated with PEO:$CsClO_4$

The Hall coefficient, $R_H = V_{12eff}/(I_D B) = 1/(qn_s)$ in cm^2/C was calculated using the effective Hall voltage V_{12eff}, magnetic field, B, and elementary charge, q. The average n_s as a function of V_{SG} is reported in Figure 2a, 4e and 6b. The standard deviation in V_{12} measured at both B = 0 and ±1 T is propagated using the rules of propagation of uncertainties at every step and the final uncertainty in n_s is reported as error bars.

Time-dependent V_{12} for three different electrolyte thicknesses at $V_{SG} = \pm 3$ V is repre-

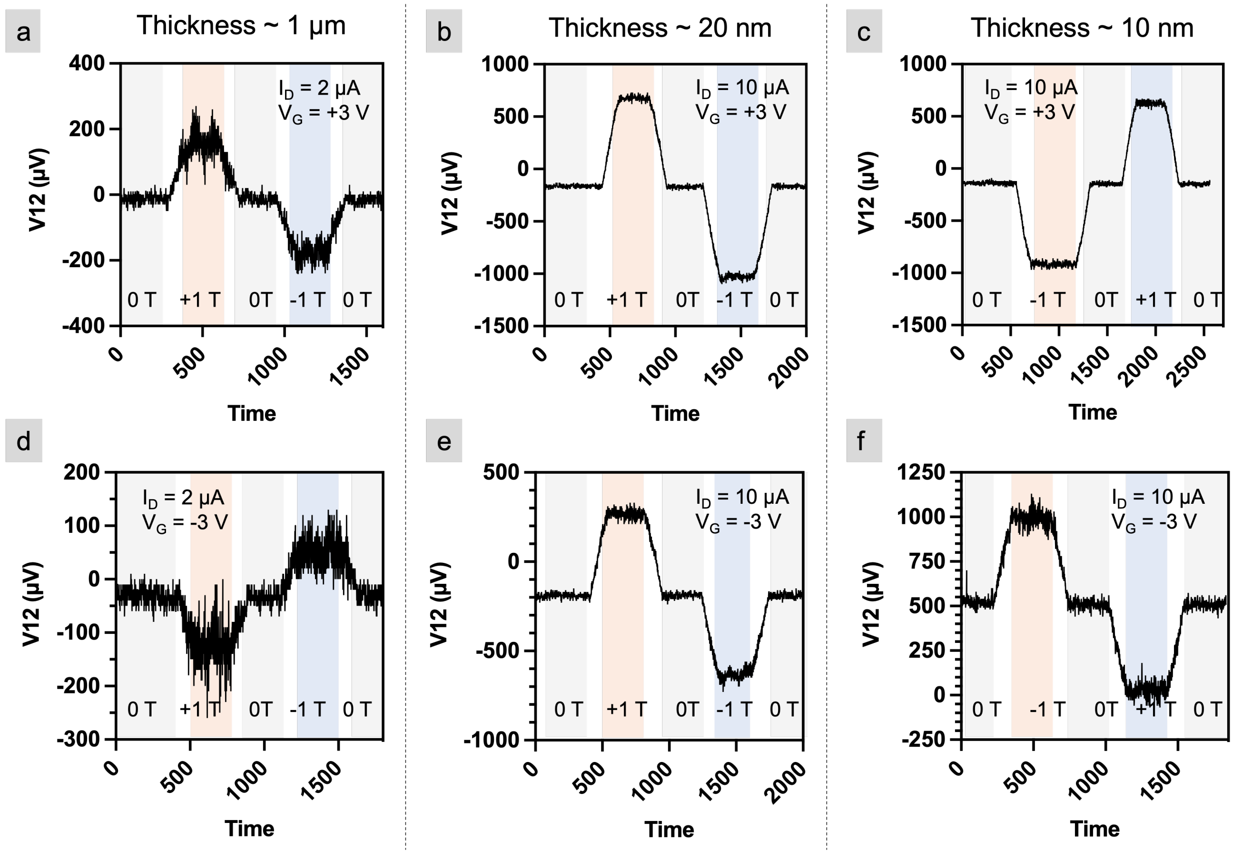

Figure S5: Time-dependent Hall voltage (V_{12}) using (a and d) bulk ($\sim$ 1 μm), (c and e) 20 and (d and f) 10 nm PEO:$CsClO_4$ at V_{SG} = +3 and −3 V, respectively.

sented in Figure S5. The reason for large error bars when using bulk ($\sim$ 1 μm) electrolyte is because of the larger noise in the V_{12} measurements (Figures S5a and d). The Hall measurement using bulk electrolyte was done by applying I_D = 2 μA (vs. 10 μA for 10 and 20 nm electrolyte) which generates Hall voltages approaching the measurement limit of the instrument ($\sim$100s μV). In contrast, because of the higher current, Hall voltages generated for 10 and 20 nm electrolyte are comparatively larger (> 500 μV).

References

(1) Kim, S. H.; Hong, K.; Xie, W.; Lee, K. H.; Zhang, S.; Lodge, T. P.; Frisbie, C. D. Electrolyte-Gated Transistors for Organic and Printed Electronics. *Advanced Materials* **2013**, *25*, 1822–1846.

(2) Efetov, D. K.; Kim, P. Controlling electron-phonon interactions in graphene at ultrahigh carrier densities. *Physical Review Letters* **2010**, *105*, 256805.

(3) Ye, J.; Craciun, M. F.; Koshino, M.; Russo, S.; Inouea, S.; Yuan, H.; Shimotani, H.; Morpurgo, A. F.; Iwasa, Y. Accessing the transport properties of graphene and its multilayers at high carrier density. *Proceedings of the National Academy of Sciences of the United States of America* **2011**, *108*, 13002–13006.

(4) Li, H. M.; Xu, K.; Bourdon, B.; Lu, H.; Lin, Y. C.; Robinson, J. A.; Seabaugh, A. C.; Fullerton-Shirey, S. K. Electric Double Layer Dynamics in Poly(ethylene oxide) LiClO4 on Graphene Transistors. *Journal of Physical Chemistry C* **2017**, *121*, 16996–17004.

(5) Piatti, E.; De Fazio, D.; Daghero, D.; Tamalampudi, S. R.; Yoon, D.; Ferrari, A. C.; Gonnelli, R. S. Multi-Valley Superconductivity in Ion-Gated MoS2 Layers. *Nano Letters* **2018**, *18*, 4821–4830.

(6) Saito, Y.; Nakamura, Y.; Bahramy, M. S.; Kohama, Y.; Ye, J.; Kasahara, Y.; Nakagawa, Y.; Onga, M.; Tokunaga, M.; Nojima, T.; Yanase, Y.; Iwasa, Y. Superconductivity protected by spin-valley locking in ion-gated MoS2. *Nature Physics* **2016**, *12*, 144–149.

(7) Zheliuk, O.; Lu, J. M.; Chen, Q. H.; Yumin, A. A.; Golightly, S.; Ye, J. T. Josephson coupled Ising pairing induced in suspended MoS2 bilayers by double-side ionic gating. *Nature Nanotechnology* **2019**, *14*, 1123–1128.

(8) Shi, W.; Ye, J.; Zhang, Y.; Suzuki, R.; Yoshida, M.; Miyazaki, J.; Inoue, N.; Saito, Y.; Iwasa, Y. Superconductivity Series in Transition Metal Dichalcogenides by Ionic Gating. *Scientific Reports* **2015**, *5*, 12534.

(9) Zhang, H.; Berthod, C.; Berger, H.; Giamarchi, T.; Morpurgo, A. F. Band Filling and Cross Quantum Capacitance in Ion-Gated Semiconducting Transition Metal Dichalcogenide Monolayers. *Nano Letters* **2019**, *19*, 8836–8845.

(10) Xu, H.; Fathipour, S.; Kinder, E. W.; Seabaugh, A. C.; Fullerton-Shirey, S. K. Reconfigurable ion gating of 2H-MoTe2 field-effect transistors using poly(ethylene oxide)-CsClO4 solid polymer electrolyte. *ACS Nano* **2015**, *9*, 4900–4910.

(11) Allain, A.; Kis, A. Electron and hole mobilities in single-layer WSe2. *ACS Nano* **2014**, *8*, 7180–7185.

(12) Robitaille, C.; Fauteux, D. Phase Diagrams and Conductivity Characterization of Some PEO-LiX Electrolytes. *Journal of The Electrochemical Society* **1986**, *133*, 315–325.

(13) Fullerton-Shirey, S. K.; Maranas, J. K. Effect of LiClO 4 on the structure and mobility of PEO-based solid polymer electrolytes. *Macromolecules* **2009**, *42*, 2142–2156.

(14) Halim, S. I. A.; Chan, C. H.; Kressler, J. Effects on the properties after addition of lithium salt in poly(Ethylene oxide)/poly(methyl acrylate) blends. *Polymers* **2020**, *12*, 2963.
